# Influence of mutation rate on estimators of genetic differentiation - lessons from *Arabidopsis thaliana*

**DOI:** 10.1186/1471-2156-11-33

**Published:** 2010-05-01

**Authors:** Ilkka Kronholm, Olivier Loudet, Juliette de Meaux

**Affiliations:** 1 Department of Plant Breeding and Genetics, Max-Planck Institute for Plant Breeding Research, Carl-von-Linné-Weg 10, 50829 Cologne, Germany; 2 Institut Jean-Pierre Bourgin, UMR1318 INRA-AgroParisTech, F-78000 Versailles, France

## Abstract

**Background:**

The analysis of molecular variation within and between populations is crucial to establish strategies for conservation as well as to detect the footprint of spatially heterogeneous selection. The traditional estimator of genetic differentiation (*F*_*ST*_) has been shown to be misleading if genetic diversity is high. Alternative estimators of *F*_*ST *_have been proposed, but their robustness to variation in mutation rate is not clearly established. We first investigated the effect of mutation and migration rate using computer simulations and examined their joint influence on *Q*_*ST*_, a measure of genetic differentiation for quantitative traits. We further used experimental data in natural populations of *Arabidopsis thaliana *to characterize the effect of mutation rate on various estimates of population differentiation. Since natural species exhibit various degrees of self-fertilisation, we also investigated the effect of mating system on the different estimators.

**Results:**

If mutation rate is high and migration rate low, classical measures of genetic differentiation are misleading. Only Φ_*ST*_, an estimator that takes the mutational distances between alleles into account, is independent of mutation rate, for all migration rates. However, the performance of Φ_*ST *_depends on the underlying mutation model and departures from this model cause its performance to degrade. We further show that *Q*_*ST *_has the same bias. We provide evidence that, in *A. thaliana*, microsatellite variation correlates with mutation rate. We thereby demonstrate that our results on estimators of genetic differentiation have important implications, even for species that are well established models in population genetics and molecular biology.

**Conclusions:**

We find that alternative measures of differentiation like *F'*_*ST *_and *D *are not suitable for estimating effective migration rate and should not be used in studies of local adaptation. Genetic differentiation should instead be measured using an estimator that takes mutation rate into account, such as Φ_*ST*_. Furthermore, in systems where migration between populations is low, such as *A. thaliana*, *Q*_*ST *_<* F*_*ST *_cannot be taken as evidence for homogenising selection as has been traditionally thought.

## Background

Characterisation of population structure is a pivotal task in population genetics. It is important for inferring the evolutionary history of a species, assisting in conservation studies [1] and measuring dispersal [2-4]. In association mapping and forensic applications, population structure must be corrected for [see [5]]. The characterisation of population structure is also crucial for studies tracking the signature of local adaptation i. e. when natural selection maintains differences in allele frequencies between populations. Indeed, limited migration or population bottlenecks can also cause allele frequencies to diverge. A proper characterisation of population structure is absolutely required to infer the action of local selection on outlier loci with reasonable confidence [6].

One way to quantify population structure is to use the summary statistic *F*_*ST*_, which measures population differentiation. If allele frequencies are similar in a set of populations *F*_*ST *_will be low, and conversely, if allele frequencies are different *F*_*ST *_will be high. Basically, *F*_*ST *_and its hierarchical extensions quantify how genetic diversity is partitioned within and between populations or groups of populations (see [5,7-9] for a review of the large body of theory on *F*_*ST*_). *F*_*ST *_can also be defined in terms of the ratio of coalescence times, for pairs of alleles from the same population over pairs of alleles from different populations [7]. If we consider only identity by descent, the topology of the genealogy is independent of the number of mutations.

*F*_*ST *_can be sometimes related to quantities describing migration [2], although this approach has its caveats [4]. In the context of selection, by using many presumably neutral markers one can build a distribution of expected *F*_*ST *_values and then compare these to *F*_*ST *_values of genes that are hypothesised to be subject to selection [9]. This can also be done for phenotypes using *Q*_*ST*_, a measure of genetic differentiation in quantitative traits. It has been shown that under neutrality *Q*_*ST *_is equivalent to a single locus *F*_*ST*_, and this relationship is robust to different demographic scenarios [10-12]. This property can be used to compare the *Q*_*ST *_of a quantitative trait to the distribution of *F*_*ST *_from neutral markers and infer whether the trait is influenced by selection [reviewed in [13]].

Some recent studies have raised concerns about the reliability of *F*_*ST *_for characterisation of population structure using markers with high mutation rates, such as microsatellites [14-18]. High levels of within population diversity bias *F*_*ST *_estimates downwards, because *F*_*ST *_is estimated using heterozygosities or genetic variances. If a locus has multiple alleles, classical *F*_*ST *_can be low even if populations share no alleles [15,16,19]. In addition to the classical *F*_*ST*_, there are other estimators that have been proposed over the years. An analogous estimator to *F*_*ST*_, Φ_*ST*_, takes into account the distances between alleles thereby correcting for mutation rate [7,20]. Classical *F*_*ST*_, estimated in the framework of Weir & Cockerham [21] considers only allele identity while Φ_*ST *_considers distances between the alleles, be it differences in repeat number (e.g. in the case of microsatellite) or number of pairwise differences between DNA haplotypes. Another measure, *F'*_*ST*_, standardises the observed *F*_*ST *_value with the maximum possible value that *F*_*ST *_could attain given the amount of observed diversity [15]. Finally, Jost derived recently a new measure of genetic differentiation, *D *[16] to replace *G*_*ST *_(or its equivalent for empirical studies *F*_*ST*_). *D *measures allelic differentiation by partitioning heterozygosity into within and between population components [16]. *D *is defined as , where *H*_*T *_is total heterozygosity, *H*_*S *_subpopulation heterozygosity and *n *is the number of subpopulations [16]. These estimators provide improved measures of allelic differentiation between populations, yet their usefulness for estimating genetic differentiation in the context of migration or local adaptation is not clearly established. Most previous studies addressed these concerns using *G*_*ST *_instead of *F*_*ST*_. When *G*_*ST *_is used, the mathematics are less complicated. In practise, however, *F*_*ST *_has better statistical properties when empirical studies are concerned [see [5]]. For this reason, *F*_*ST *_is the statistic of interest in this study. Moreover, in practise the difference between these two statistics is small.

Here, we characterize the relationship between different estimators of genetic differentiation and mutation rate using simulations as well as experimental data collected from natural populations. Our study system is *Arabidopsis thaliana *(L.) Heyhn. (Brassicaceae). We use simulations to characterize the relationship between mutation rate and population differentiation for both molecular markers and quantitative traits and ask the following questions: i) are all estimators of genetic differentiation dependent on mutation rate, ii) is the estimator of differentiation for quantitative traits dependent on mutation rate, iii) does the mating system influence the relationship between mutation rate and estimators of population differentiation. We provide empirical data from *Arabidopsis thaliana*, a long-standing model for plant molecular biology that has recently become a model in plant population genetics [22]. We show that in *A. thaliana*, *F*_*ST *_is biased downwards for high mutation rate loci. We discuss these results and their implications for various applications of *F*_*ST *_analysis, including the reliable detection of variable selection.

## Methods

### Data analysis - Computer simulations

In order to investigate the behaviour of *F*_*ST*_, *F'*_*ST*_, Φ_*ST *_and *D *under high mutation rates, computer simulations using EasyPop 1.8 [23] were performed. The simulation scheme was set to 10 populations with 500 individuals each, 20 freely recombining loci and random mating hermaphrodites. All loci were set initially as polymorphic by allowing EasyPop to generate the genotypes in the first generation. Populations followed an island model of migration. Migration rates (probability that a given individual will migrate in each generation), *m *ranged from 0.1 to 0.00001 and mutation rates (probability that a given allele will mutate in each generation), *μ *from 0.00001 to 0.01. In order to simulate microsatellite loci we first examined a pure single step mutation model. Then we relaxed this assumption by using a mixed mutation model in which the loci followed a single step mutation model but with the probability of 0.2 to mutate to any state. The number of possible allelic states was set to 30. The effect of self-fertilisation was examined by doing simulations with proportion of self-fertilisation set to 0.9. Simulations were run for 2000 generations. In our simulations this was enough for *F*_*ST *_to reach equilibrium. To simulate a realistic sampling situation, 30 individuals were finally sampled from each population for parameter estimation. Each simulation was repeated 5 times for a given set of parameter values. For each simulated dataset we calculated the estimators of genetic differentiation as described below.

Next we examined how mutation rate at underlying QTL affects *Q*_*ST*_. We used quantiNEMO [24], with the same settings as described above for neutral markers with the following exceptions: the number of QTL underlying the variation in the quantitative trait was 10 and there were 21 possible allelic states for each QTL. We used the random mutation model in quantiNEMO for the QTL alleles, in this model, allelic effects are drawn from a normal distribution, all effects are additive in our simulations. We also ran the simulations using the incremental mutation model, where the allelic effect of a new mutation resembles its ancestor. Variance of allelic effects was set to 0.1. The simulated quantitative trait was neutral and did not have any effect on fitness. The simulation was started at a state where all loci were monomorphic, the number of generations was 4000. The time to reach equilibrium was longer for low migration and mutation rates and in these cases number of generations was 6000. This is longer than for neutral markers because simulations had to be started from a monomorphic state; otherwise distribution of allelic effects becomes unrealistic. Variance components for *Q*_*ST *_were estimated from genotypic values, which are returned by quantiNEMO as output, using R-scripts written by IK. The statistical model was a mixed-effect model with populations as random factors; REML-estimates of variance components were used. This was done in order to calculate *Q*_*ST *_also in the presence of self-fertilisation, which quantiNEMO does not calculate as a standard output. *Q*_*ST *_was estimated from the equation [25], where  is the between populations genetic variance component,  is the within populations genetic variance component and *F*_*IS *_is reduction of heterozygosity within individuals due to inbreeding. Our method of estimating *Q*_*ST *_gives the same results as the standard output of quantiNEMO when mating is random (results not shown). For each parameter set, 50 replicates of simulations with a single quantitative trait were run.

We also performed coalescent simulations to investigate the effect of different marker types on *F*_*ST *_calculations. We investigated DNA haplotypes (these would be derived by re-sequencing short fragments, one locus is one fragment), independent single SNP markers and microsatellite markers following a single step mutation model. All coalescent simulations were performed using the program ms [26]. We simulated an island model of population structure with 10 populations, 20 individuals were sampled from each population. For sequence haplotypes and microsatellites 30 independent loci were simulated, for SNP markers we simulated 100 independent SNPs. For single SNPs and haplotypes, multiple hits were not permitted. The microsatellite mutation model was implemented via R-script. In the program ms migration and mutation rate are expressed in terms of effective population size, *4Nm *and *4Nμ *respectively. We set up the simulations so that the effective population size was 1000 for each population and then parameters *m *and *μ *ranged from 0.0001 to 0.1 for *m *and 0.00001 to 0.001 for *μ*. Each simulation was repeated 5 times for each parameter combination.

### Data analysis - Genetic diversity and population structure

All statistical analyses were done using the statistical environment R [27] unless otherwise stated. Methods not implemented by R-packages were implemented via R-scripts written by IK and are available upon request.

Measure of genetic diversity, Nei's gene diversity (*Hs*) was calculated using FSTAT 2.9.3 [28]. The microsatellite population mutation rate, *θ*, is the product of effective population size and mutation rate at a locus was calculated following equation 15 of Kimmel *et al*. [29]. The performance of this summary statistic based method has been shown to be comparable to likelihood-based methods [30]. *θ *was calculated for each locus within each region. For SNP data the minor allele frequency was calculated for each locus in each region.

*F*_*ST *_was estimated according to Weir & Cockerham [21] for microsatellites and SNP markers, using the R-package "hierfstat" [31]. All other genetic differentiation methods were implemented via R-scripts written by IK. For microsatellites the standardised genetic differentiation measure, *F'*_*ST *_[15], was estimated using the maximised variance component method of Meirmans [32]. In order to take the distance between the microsatellite alleles or sequence haplotypes into account [20] we estimated Φ_*ST *_using the method of Michalakis and Excoffier [33]. Differentiation indices between regions were calculated in a hierarchical setting, taking into account the partition of variation between populations within regions [7]. Confidence intervals for different measures of genetic differentiation were generated by bootstrapping over loci. An estimator for *D *was calculated following Jost [16].

The expected *F*_*ST *_was calculated for the forward and coalescent simulations using the relationship between *F*_*ST *_and coalescence times in the island model. The expected *F*_*ST *_was calculated as [7], where  = average coalescence time of alleles from different populations and  = average coalescence time of two alleles from the same population. For an island model of population structure,  and , where *N *= population size, *d *= number of populations and *m *= migration rate [34]. This provides us with an analytical estimate of the expected value of *F*_*ST*_. We used this as a baseline when comparing the different estimators.

To check whether Φ_*ST *_>*F*_*ST *_for the microsatellite loci, we used a permutation test [35]; if Φ_*ST *_>*F*_*ST *_stepwise mutations may contribute to genetic differentiation, thereby providing one explanation for the difference. The test was implemented in the program SPAGeDi 1.2 [36]. This is done by permuting microsatellite allele sizes among allelic states to test if stepwise mutations contribute to genetic differentiation.

### Population samples

In total 289 individuals from 41 populations were genotyped. Detailed information about the populations can be found in the supplementary material (Additional file 1). We analysed 7, 15, 13 and 6 populations from Spain, France, Norway and Central Asia, respectively. Number of sampled individuals from each population ranges from 3 to 11 with a mean of 7. Three regions in Western Europe: Spain, France, Norway create a South - North cline. The Central Asian region is composed of populations from Kyrgyzstan and Tajikistan. The Spanish populations are described in Pico *et al. *[37]. French populations were collected by Valerie Le Corre and some of them are described in Le Corre [38]. The Norwegian populations were kindly provided by Odd-Arne Rognli through NARC (Norway). Populations from Central Asia were collected by OL and are described at http://www.inra.fr/vast/collections.htm. Field collected plants were subjected to one or two generations of self-fertilisation in the greenhouse before DNA extraction.

### Genotyping

DNA was extracted from young leaves using BioSprint 96 robot and BioSprint 96 DNA Plant Kit (Qiagen) according to manufacturer's instructions.

Plants were genotyped at 20 microsatellite loci. Details of the microsatellite loci used and genotyping procedures can be found in the supplementary material (Additional file 2). Microsatellites were amplified using standard PCR methods and allele sizes were determined using capillary electrophoresis. To determine the actual number of repeats in each allele, the accession Col-0 was genotyped for each locus as a reference. Using the genome sequence and the Col-0 PCR product size the actual number of repeats was deduced for each allele. The Spanish accessions had already been genotyped previously for some of the loci used here, as described in Pico *et al. *[37]. We verified that our allele sizes corresponded to the allele sizes reported previously by re-genotyping a subsample at selected alleles.

The plants were also genotyped for a set of 149 single nucleotide polymorphism (SNP) markers [developed by [39]] by Sequenom, inc. (San Diego, CA). Detailed description of the SNP markers is found in supplementary material (Additional file 3). Out of the 149 SNP markers, 12 loci had to be excluded because the genotyping failed or because there was no variation, leaving 137 loci used in the analysis. Microsatellite and SNP genotypes can be found in supplementary material (Additional file 4)

### Data analysis - SNP ascertainment bias

The SNP markers we used were discovered in the study of Nordborg et al. [40] and subsequently developed for mapping purposes [39]. The criterion for ascertainment was that one allele of a SNP had to be carried by the standard laboratory accession Col-0 and the other allele had to be in high frequency in the rest of the sample. Hereafter we refer to the publicly available dataset of Nordborg et al. as Nordborg data. To investigate the ascertainment bias in our sample we calculated minor allele frequency distribution for all of the SNPs in the Nordborg data and the 137 SNPs used in this study. Because the sample in which the SNPs were discovered is different from the sample in this study ascertainment bias cannot be corrected in a straightforward manner. Therefore, we examined the effect of ascertainment bias on *F*_*ST *_in the dataset from which they were selected. We used the 137 SNPs used in our study to calculate *F*_*ST *_between genetic clusters defined by Nordborg *et al. *[40] in the Nordborg dataset. Then we sampled 137 SNPs at random from the Nordborg dataset 1000 times and calculated *F*_*ST *_between the genetic clusters for each sampled dataset.

## Results

### Computer simulations

We used forward population genetic simulations to investigate the behaviour of different estimators with varying migration and mutation rates. The best estimator, in the context of local adaptation, should be robust to mutation rate to allow comparisons between different marker types.

**Figure 1:**
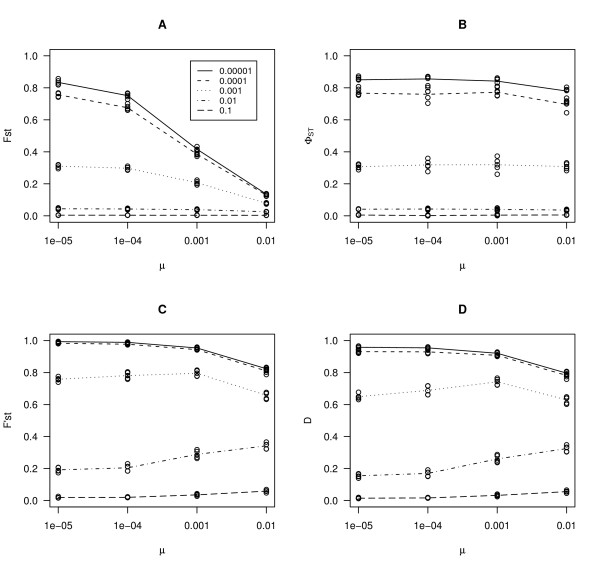
**Results of computer simulations for single step mutation model**. Different estimators of genetic differentiation were plotted against mutation rate. Different lines represent different migration rates. Migration rates 0.1, 0.01, 0.001, 0.0001 and 0.00001 correspond to different lines as indicated by the legend in panel A. Different estimators are *F*_*ST*_, Φ_*ST*_, *F'*_*ST *_and *D *in panels A, B, C and D respectively.

**Figure 2:**
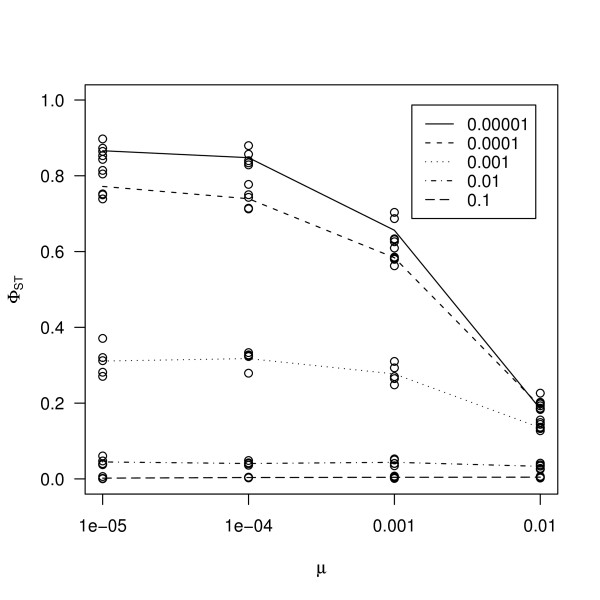
**Results of computer simulations for mixed mutation model**. The effect of mutation rate on genetic differentiation calculated from Φ_*ST *_using mixed mutation model. In this model, there was a probability of 0.2 that when a mutation occurs the allele will mutate to any state. Different lines represent different migration rates. Migration rates 0.1, 0.01, 0.001, 0.0001 and 0.00001 correspond to different lines as indicated by the legend.

For a low mutation rate, *F*_*ST *_and Φ_*ST *_were reasonably good, but *F'*_*ST *_and *D *differed from the expected value (Table 1), although for extremely small migration rates the situation was reversed. Results of forward population genetic simulations showed that *F*_*ST *_tended to zero when mutation rate increased (Figure 1). Replicate simulations clustered very well showing that there was little variance among replicates. These results follow the analytical expectation presented in Hedrick [15] and the results of Balloux & Goudet [18]. If mutations followed a pure single step model, Φ_*ST *_was essentially independent from mutation rate (Figure 1) as expected; this is true for all migration rates. *F'*_*ST *_and *D *are not independent from mutation rate. In our simulations, we observed that when migration rate was very low, increasing mutation rate up to 0.01 also caused *F'*_*ST *_and *D *to go downward (Figure 1, panels C and D). If the assumptions of single step mutation model were relaxed, Φ_*ST *_had the same trend as *F*_*ST *_although the effect was somewhat weaker (Figure 2). A self-fertilisation rate of 0.9 did not qualitatively alter the results (Additional file 5). We note that, when mutation rate was increased up to 0.01, all estimators decreased (Figure 1). This is not a cause for concern, since this rate is biologically unrealistic. This effect is explained by the limited number of alleles in the simulations, which at high mutation rate can cause homoplasy.

**Table 1:** Comparison of expected and observed values for *F*_*S*__*T*_

Forward simulations					
m	E(*F*_*ST*_)	O(*F*_*ST*_)	O(Φ_*ST*_)	O(*F'*_*ST*_)	O(*D*)
0.1	0.0045	0.0040	0.0049	0.0190	0.0148
0.01	0.0431	0.0440	0.0414	0.1920	0.1549
0.001	0.3104	0.3104	0.3067	0.7581	0.6492
0.0001	0.8182	0.7570	0.7667	0.9831	0.9307
0.00001	0.9783	0.8339	0.8500	0.9931	0.9580

**Coalescent simulations**					
**m**	**E(*F*_*ST*_)**	**O(SNP *F*_*ST*_)**	**O(MSAT Φ_*ST*_)**		**O(DNA Φ_*ST*_)**

0.1	0.0023	0.0277	0.0287		0.0299
0.01	0.0220	0.0400	0.0420		0.0488
0.001	0.1837	0.2057	0.2003		0.2127
0.0001	0.6923	0.6950	0.7124		0.6933

Expected *F*_*ST *_was calculated as described in the methods, observed values are the simulation means for the different estimators or marker types. For the coalescent simulations MSAT means microsatellite markers and DNA means DNA sequence haplotypes. Simulation values are shown for a single mutation rate, *μ *= 0.00001.

**Figure 3:**
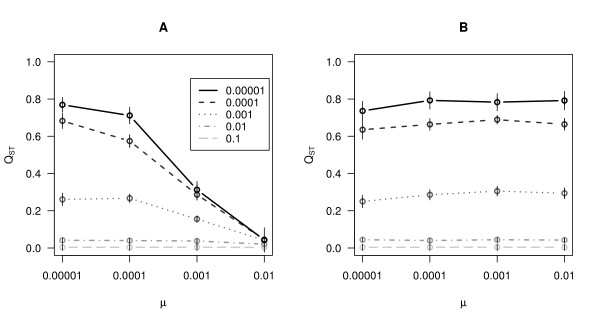
**Results for computer simulations for *Q*_*ST*_**. The effect of mutation rate and migration rate on *Q*_*ST*_. Different lines represent different migration rates. Migration rates 0.1, 0.01, 0.001, 0.0001 and 0.00001 correspond to different lines as indicated by the legend in panel A. Points are means of 50 replicate simulations and vertical lines correspond to 95% confidence intervals. In panel A) results for the random mutation model, panel B) results for the incremental mutation model.

We then examined the effect of mutation rate at QTL on *Q*_*ST*_. We observed the same behaviour as for *F*_*ST*_. When migration rates are low, increasing the mutation rate at underlying QTLs caused *Q*_*ST *_to take lower values (Figure 3). We also performed the simulations using an increment mutation model for the QTL, where each new mutation had an allelic value that is close to the allelic value of its ancestor. In this case *Q*_*ST *_behaved like Φ_*ST *_and was not affected by mutation rate (Figure 3, panel B). Here again, a self-fertilisation rate of 0.9 did not qualitatively alter the results (Additional file 6).

**Figure 4:**
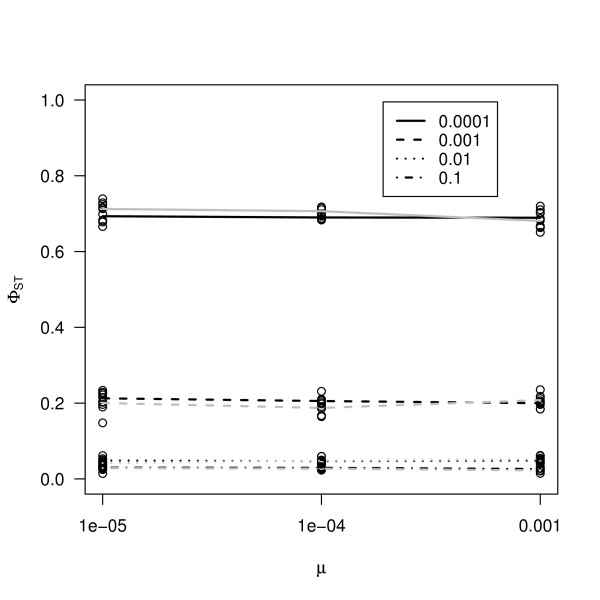
**Results of coalescent simulations for different marker types**. The effect of mutation rate on genetic differentiation, calculated from Φ_*ST*_. Black lines represent estimates from DNA haplotypes, grey lines are estimates from microsatellite alleles. Different line types represent different migration rates. Migration rates 0.1, 0.01, 0.001 and 0.0001 correspond to different lines as indicated by the legend.

Next we examined the effect of mutation rate on different marker types. We simulated DNA haplotypes (as would be derived by re-sequencing short fragments from multiple individuals), microsatellite markers and single SNP markers. Results from the simulations are presented in figure 4. We calculated Φ_*ST *_that takes into account distance between different haplotypes or microsatellite alleles. Applying this method to both haplotypes and microsatellites gave essentially the same results (Figure 4 and Table 1) and Φ_*ST *_was independent from mutation rate for both marker types. Single SNP markers also gave *F*_*ST *_values that were nearly identical to the ones obtained with other types of markers (Table 1). This is in accordance with DNA haplotype and microsatellite markers (Figure 4). Therefore, Φ_*ST *_for DNA haplotypes, microsatellites (following single step mutation model) and *F*_*ST *_for single SNPs (free of ascertainment bias) gave comparable estimates of differentiation.

### Genetic differentiation in *Arabidopsis thaliana*

**Figure 5:**
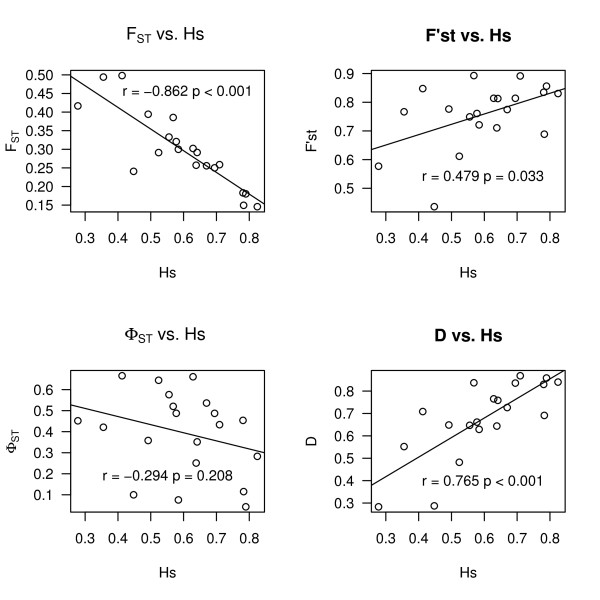
**Correlations between genetic diversity and genetic differentiation in Spanish populations**. Gene diversity, Hs, was calculated for each locus and was plotted against different estimators of genetic differentiation. A) *F*_*ST *_B) *F'*_*ST *_C) Φ_*ST *_D) *D*

We characterised diversity within 41 populations distributed in four broad geographic regions. We observed that genetic differentiation (*F*_*ST*_) for microsatellite loci correlates with gene diversity (*Hs*) (Figure 5). For instance, in the Spanish populations the correlation between *Hs *and *F*_*ST *_was r = -0.862 (95% CI = -0.944 - -0.678) with p < 0.001 (Table 2). We further examined the correlation between diversity and various alternative estimators of differentiation. There was positive albeit non-linear relationship between *Hs *and *F'*_*ST*_, (r = 0.479, [95% CI = 0.076 - 0.760], p = 0.033). Φ_*ST *_was not correlated with *Hs*, (r = -0.294, [95% CI = -0.652 - 0.170], p = 0.208). A similar pattern was observed when the population mutation rate (*θ*) was used instead of *Hs*. For *θ *and *F*_*ST *_r = -0.682, p < 0.001, for *θ *and *F'*_*ST *_r = 0.500, p = 0.025 and for *θ *and Φ_*ST *_r = -0.301, p = 0.197, respectively. Φ_*ST *_is independent from genetic diversity and mutation rate in our data, except in Central Asian populations (Table 2, Figure 5).

**Table 2:** Correlations between genetic diversity and genetic differentiation

	*Hs*		*θ*	
Spanish populations	r (95% CI)	p	r (95% CI)	p
*F*_*ST*_	-0.862 (-0.944 - -0.678)	<0.001	-0.682 (-0.864 - -0.342)	<0.001
*F'*_*ST*_	0.479 (0.046 - 0.760)	0.033	0.500 (0.074 - 0.771)	0.025
Φ_*ST*_	-0.294 (-0.652 - 0.170)	0.208	-0.301 (-0.656 - 0.163)	0.197
*D*	0.765 (0.488 - 0.902)	<0.001	0.655 (0.300 - 0.851)	0.002

French populations				
*F*_*ST*_	-0.867 (-0.948 - -0.681)	<0.001	-0.625 (-0.840 - -0.238)	0.004
*F'*_*ST*_	0.645 (0.270 - 0.850)	0.003	0.705 (0.370 - 0.878)	<0.001
Φ_*ST*_	-0.260 (-0.639 - 0.220)	0.282	-0.144 (-0.561 - 0.332)	0.557
*D*	0.876 (0.700 - 0.952)	<0.001	0.756 (0.460 - 0.901)	<0.001

Norwegian populations				
*F*_*ST*_	-0.916 (-0.968 - -0.791)	<0.001	-0.599 (-0.828 - -0.198)	0.007
*F'*_*ST*_	0.199 (-0.280 - -0.599)	0.413	0.446 (-0.011 - 0.748)	0.056
Φ_*ST*_	-0.109 (-0.536 - 0.364)	0.658	-0.021 (-0.471 - 0.437)	0.931
*D*	0.631 (0.248 - 0.843)	0.004	0.717 (0.390 - 0.0.884)	<0.001

Central Asian populations				
*F*_*ST*_	-0.801 (-0.928 - -0.506)	<0.001	-0.494 (-0.795 - 0.002)	0.052
*F'*_*ST*_	-0.116 (-0.578 - 0.403)	0.669	0.125 (-0.395 - 0.584)	0.645
Φ_*ST*_	-0.628 (-0.857 - -0.192)	0.009	-0.132 (-0.589 - 0.389)	0.625
*D*	0.565 (-0.013 - 0.860)	0.055	0.484 (-0.124 - 0.828)	0.111

Correlations between genetic diversity in markers and genetic differentiation between populations in different regions. Correlation coefficients are given with 95% confidence intervals are in parenthesis. *Hs *is subpopulation heterozygosity and *θ *is an estimate of microsatellite mutation rate.

The 137 polymorphic SNP markers used in this study were biased towards high frequency as shown in Additional file 7, where minor allele frequency is plotted for each SNP in different regions. This was apparent also from comparing the minor allele frequency spectrum of the full Nordborg data and the 137 SNP set (Additional file 7). Using the 137 SNP set we obtained *F*_*ST *_= -0.0018 between clusters defined previously in the Nordborg data [40]. Sampling 137 SNPs at random from the Nordborg data gave us a 95% confidence interval of *F*_*ST *_-0.0051 - 0.0271. This suggests that ascertainment bias perhaps has only a minor effect in our dataset.

**Figure 6:**
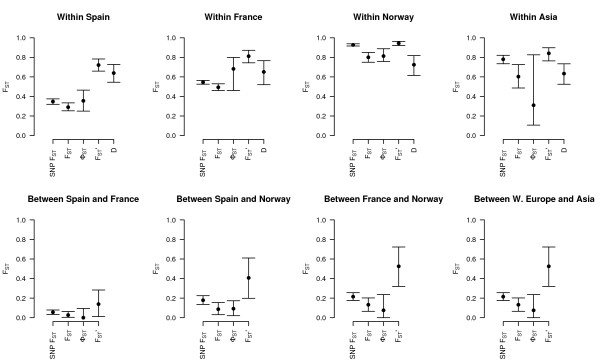
**Genetic differentiation within and between regions**. Genetic differentiation between populations within regions and between regions. Differentiation was estimated from microsatellite markers using four different estimators (for explanation, see methods). Points are estimates of differentiation over all loci, bars represent its 95% CI, obtained by bootstrapping over loci. For SNP markers *F*_*ST *_was used. Between group values were not calculated for *D*, because as of yet *D *has not been hierarchically extended.

Next we calculated measures of genetic differentiation for microsatellites and SNP markers between populations within regions and between regions (Figure 6). For microsatellites, genetic differentiation between populations was the lowest in Spain (*F*_*ST *_= 0.2900, Φ_*ST *_= 0.3556), intermediate for France (*F*_*ST *_= 0.4937, Φ_*ST *_= 0.6818) and for Asia (*F*_*ST *_= 0.6026, Φ_*ST *_= 0.3101) and the highest in Norway (*F*_*ST *_= 0.8004, Φ_*ST *_= 0.8128). A similar trend was observed for both microsatellites and SNP markers (Figure 6). However, it should be noted that the confidence intervals were sometimes broad (Figure 6), especially in Central Asia. Genetic differentiation between geographic regions was smaller than between populations within regions (Figure 6). Differentiation measured by *F'*_*ST *_from microsatellites was *F'*_*ST *_= 0.7208 for Spain, 0.8115 for France, 0.9436 for Norway and 0.8413 for Asia. Values for *D *were 0.6393, 0.6509, 0.7241 and 0.6334 for the Spanish, French, Norwegian and Cental Asian populations respectively.

We tested whether Φ_*ST *_was higher than *F*_*ST *_by using a permutation test that permutes allele sizes between different alleles [35]. If Φ_*ST *_is higher than *F*_*ST*_, one possibility is that stepwise mutations contribute to differentiation [35]. Within Spanish populations, the difference is suggestive albeit not significant, 2-sided test p = 0.0629. Within French populations, the difference was significant p = 0.0210. In Norwegian and Asian populations, differences were not significant (p = 0.1009 and p = 0.8561 respectively). This suggests that stepwise mutations may contribute to genetic differentiation in Spain and France. In the Norwegian and Asian populations instead, microsatellite loci possibly exhibit some departure from a stepwise mutation model. However, this does not prove that the loci follow SSM model.

## Discussion

### Implications for detecting local adaptation

We used computer simulations to analyze the behaviour of different estimators of genetic differentiation as a function of various migration and mutation rates. Our simulations show that *D *or *F'*_*ST *_both depend on mutation rate (Figure 1). For *D*, this is lucidly shown by Jost [16] but the fact that *F'*_*ST *_is dependent on mutation rate is not made clear by Hedrick [15]. New mutations increase differentiation between populations, especially if migration rate is low. *D *and *F'*_*ST *_measure actual allelic differentiation, regardless of the process that generates these differences [41]. This means that *F'*_*ST *_or *D *are useful for studies where the amount of genetic differentiation is of interest *per se*, such as in conservation studies [15,16]. Instead, these estimators become problematic for studies interested in comparing levels of differentiation across loci, as in studies of local adaptation. Also, they cannot be directly related to coalescence times (Table 1). This is not necessarily a criticism of *F'*_*ST *_and *D*, since *D *was derived to measure true allelic differentiation. These measures address the question of the partition of genetic diversity among populations, irrespective of the mechanisms by which this pattern arises [16]. Further work is needed to characterise the properties of these estimators.

Our results show that, because it takes distances between different alleles into account, Φ_*ST *_is the only estimator that is completely independent from mutation rate (Figure 1) [18,20]. We also showed that if the assumptions of Φ_*ST *_are met, both DNA haplotypes and microsatellites give estimates comparable to *F*_*ST *_calculated for bi-allelic SNPs (Figure 4). In a large empirical dataset in humans [42], where microsatellites analysed using a stepwise mutation model and SNPs gave comparable results, a similar conclusion was reached. In *A. thaliana *as in many other species, microsatellite loci were often shown to deviate from pure single step mutation model [43-46]. Our simulations further show that this estimator became dependent on mutation rate, if there was some deviation from the single step mutation model (Figure 2). This is also seen in our dataset in some geographic regions, as we find that Φ_*ST *_is not different from *F*_*ST*_. Yet, Φ_*ST *_might still be preferred over *F*_*ST*_. Even if there are deviations from the SMM model, Φ_*ST *_still performs better than *F*_*ST *_(Figure 1, Figure 2).

SNP markers are unlikely to suffer from these issues in *F*_*ST *_estimation because mutation rate for SNP markers is considerably lower than for microsatellites. However, they may have some ascertainment bias. This effect, however, was minor in the 96 genotypes in which our SNP-marker set was ascertained. Although it is not certain that this behaviour would be the same in our set of 41 populations, estimates of differentiation based on SNPs are roughly concordant with Φ_*ST *_estimates based on microsatellites (Figure 6). In Norway and central Asia however, microsatellite Φ_*ST *_seems to be lower than *F*_*ST *_for SNP markers (Figure 6). In these regions, we do not have any evidence that microsatellites follow a stepwise mutation model, so this seems a likely cause for the discrepancy. However, ascertainment bias cannot be completely excluded. In many cases, confidence intervals for Φ_*ST *_are broad in our data, a likely consequence of the high sampling variance displayed by Φ_*ST *_type estimators [47]. This consideration suggests that, whenever possible, DNA haplotypes generated by re-sequencing should be used in studies of local adaptation.

It is known that there is considerable variation in mutation rates between different genes due to evolutionary constraints [48]. In order to directly compare differentiation across genes and correctly assign loci subject to local adaptation, mutation rate has to be taken into account. This problem has been considered earlier in the context of detecting loci that have outlier *F*_*ST *_values [49]. The method of Beaumont & Nichols jointly considers heterozygosity and *F*_*ST*_. This method was shown to be robust to mutation rate variation among loci [49]. Yet if the types of markers combined in one analysis are different and mutation rate has a strong influence for one type of marker because of low migration rates, results have to be interpreted with caution. Indeed, this method gave non sensical results with our *A. thaliana *dataset combining microsatellite and SNP markers (data not shown).

Our results have further implications for studies based on *Q*_*ST *_vs. *F*_*ST *_comparisons, aiming at detecting local adaptation in quantitative traits. A recent meta-analysis of *F*_*ST *_vs. *Q*_*ST *_studies [50] noted that using *F'*_*ST *_would generally change the conclusions of *F*_*ST *_vs. *Q*_*ST *_studies. However, our study shows that using *F'*_*ST *_or *D *in *Q*_*ST *_studies is not appropriate, because these measures of genetic differentiation are not independent from the high mutation rate of microsatellites. Yet, this is a concern only for those cases where migration rate is low.

Another concern is the fact that *Q*_*ST*_, the estimate for quantitative traits, is affected by the mutation rate at underlying QTLs (Figure 3). In retrospect, *Q*_*ST *_being an equivalent of *F*_*ST *_in the absence of selection [10], this result is not completely surprising. More interesting however, is the parameter range at which this bias is observed. In our simulations we varied the parameter *μ*, the genic mutation rate. It should be noted that the overall polygenic mutation rate, or variance contributed by mutation in each generation (assuming additive effects), of a quantitative trait is *V*_*m *_= 2 *μN*_*L*_*V*_*α *_[51], where *N*_*L *_is the number of QTL, 2 accounts for diploidy and *V*_*α *_is the variance of allelic effects. In our simulations *N*_*L *_= 10 and *V*_*α *_= 0.1 were held constant in all simulations. This translates into *V*_*m *_of twice as large as genic mutation rate, so that for *μ *= 0.0001 *V*_*m *_= 0.0002. In our simulations environmental variance, *V*_*E*_, was set to 1 so mutational variance scaled by environmental variance, *h*^*2*^_*m *_= *V*_*m *_/ *V*_*E*_, [51,52] was equal to *V*_*m*_. In our simulations when genic mutation rate of QTL was *μ *= 0.001, effects on *Q*_*ST *_were rather large when migration rates were low (Figure 3). This corresponded to *h*^*2*^_*m *_= 0.002. It is interesting to note that empirical estimates of mutational heritabilities frequently fall around this value [52]. For instance, *h*^*2*^_*m *_for the well studied trait of bristle number in *Drosophila *seems to be around 0.0035 or 0.0043. Schultz et al. [53] estimated *h*^*2*^_*m *_for few life-history traits in *A. thaliana *and found that they fall around 0.003. Estimating mutational heritability empirically is not easy, but empirical data from several sources suggests that for many traits it is around 0.002 - 0.003 [52].

Interestingly, if new alleles display incremental changes of function, *Q*_*ST *_becomes independent of the mutation rate. However, alleles of large effect contributing to quantitative trait variation have been observed frequently in natural populations [reviewed in [54]]. A prominent example is provided by the FRIGIDA locus, a gene contributing to quantitative variation in flowering time in *A. thaliana*. Several loss-of-function mutations were reported to segregate in natural populations [38]. Thus QTL are likely to frequently deviate from a purely incremental mutation model.

A comforting fact is that high mutation rates will bias *Q*_*ST *_downwards, so if the goal is to study local adaptation, that is, the case where *Q*_*ST *_>*F*_*ST *_is of interest, then the test will remain conservative. Traditionally it was thought that *Q*_*ST *_<* F*_*ST *_would indicate the action of homogenising selection [13], that is, selection for the same phenotypic optima in different populations. Recently this view has been challenged. Firstly, non-additive gene action seems to bias *Q*_*ST *_downwards [11,55,56]. Secondly, the large variance in estimating *Q*_*ST *_and in evolutionary outcomes of single locus (trait) may be a even more serious problem [56-58]. Intuitively, systems where *F*_*ST *_is large, because migration is low, offer potentially greatest statistical power to detect *Q*_*ST *_<* F*_*ST*_. Our results instead suggest that the bias in *Q*_*ST *_may be the greatest in such cases and that the utility of *Q*_*ST *_- *F*_*ST *_comparisons may be limited to detecting diversifying selection.

Correlating estimates of population differentiation to levels of diversity has highlighted the effect of mutation rate on estimates of differentiation in *Arabidopsis thaliana. *To date, several studies have investigated patterns of population structure of *A. thaliana*, [37,59-63] but none has examined the effect of mutation rates on estimates of differentiation. We observe this effect in the four distinct geographical regions. Therefore, mutation rates seem to significantly impact estimates of population differentiation in this species. This relationship has also been found in *Arabidopsis lyrata*, a relative of *A. thaliana *exhibiting a markedly different life-history and more genetic diversity than *A. thaliana *[64,65]. Some fish species also exhibit this relationship. For example, in two subspecies of the triplefin fish, *F*_*ST *_was low and negatively correlated with expected heterozygosity (r = -0.9) [66]. Similarly O'Reilly *et al. *[67] found a relationship between heterozygosity and *F*_*ST *_in the fish walleye pollock, which they attributed to homoplasy. As shown by our simulations, this problem is more severe in systems where diversity is very high and migration between populations is low (Figure 3). These examples show that a wide variety of organisms are in the parameter space where variation in *F*_*ST *_reflects variation in both migration and mutation rates. Therefore, studies of population differentiation should systematically investigate this effect.

### Effect of the mating system

Simulations were performed under random-mating as well as with a self-fertilizing rate of 0.9 and yielded essentially the same results. From a population genetics perspective, self-fertilisation should reduce effective population size and thus coalescence times of alleles within populations [68]. This is precisely what we observe in our simulations: absolute *F*_*ST *_values are higher, but the relationship of the statistics to mutation rate remains qualitatively the same (Additional file 5). This is also true for *Q*_*ST *_(Additional file 6). Thus the effect of self-fertilisation is mainly to increase *F*_*ST *_values, but it does not alter the effect of mutation rates on its estimates. Although a rate of 0.9 is slightly lower than selfing rates reported for *A. thaliana *so far, we believe that our simulations reflect faithfully the situation in this species [59,69,70]. Indeed, there is one study that found the rate to be closer to 0.9 in one particular population [61] and recently Pico et al. [37] found rates to be variable, from 0.97 down to 0.93 in Iberian populations. Considering that some of these studies used a very limited set of markers, it is conceivable that true rates of self-fertilisation are closer to 0.9 than 0.99. This is also evident when more markers and more individuals from a single population are sampled, because much more heterozygosity is uncovered (IK, unpublished data).

## Conclusions

In this study, we lift the ambiguity lying over the effect of variable mutation rate on estimates of population differentiation and show that virtually all estimates show a dependence on higher mutation rates if migration is low. Only Φ_*ST*_, which takes mutation rate into account, controls this bias. However, in practise, Φ_*ST *_assumes a stepwise mutation model for microsatellites, which may not be correct in all cases. Several microsatellite mutation models have been proposed [46]. Future studies could examine the possibility of incorporating different models of mutation in Φ_*ST *_estimation. For this, the rules by which distance matrices between alleles or haplotypes are calculated could be modified. For sequence data, changes would be straightforward to incorporate different models of sequence evolution. More importantly, we show that *Q*_*ST *_estimates are not independent of the mutation rate of QTLs underlying quantitative traits for low migration rates. This result is obtained with a model simulating traits with realistic mutational heritabilities. Therefore *Q*_*ST *_<* F*_*ST *_cannot be interpreted as strong evidence for homogenising selection in systems where genetic differentiation is high. Our study in *A. thaliana *shows that caution is needed when tracking the signature of local adaptation on loci controlling adaptive phenotypes in this and other model species for genetics. Further studies will eventually be needed to address the properties of these various estimates in non-equilibrium populations or to investigate the impact of the genetic architecture of a trait on *Q*_*ST*_.

## Authors' contributions

The study was conceived by IK and JdM, IK performed the experiments and analysed the data. OL contributed samples. The manuscript was written by IK with input from all other authors. All authors read and approved the final manuscript.

## Supplementary Material

Additional file 1 **Population information**. An Excel file with supplementary tables for information on sampled populations. details about microsatellite and SNP loci and genotyping Click here for file

Additional file 2 **Microsatellite genotyping**. An Excel file with the microsatellite genotyping information and primer sequences. Click here for file

Additional file 3 **SNP positions**. An Excel file with the genomic positions of the SNPs used in this study. Click here for file

Additional file 4 **Genotypic data**. An Excel file with the microsatellite and SNP genotypes of the lines used in this study. Click here for file

Additional file 5 **Computer simulations with selfing**. A PDF file with a supplementary figure for results of computer simulations when the rate of self-fertilisation is set to 0.9. Click here for file

Additional file 6 **Computer simulations for *Q*_*ST *_with selfing**. A PDF file with a supplementary figure for results of computer simulations for *Q*_*ST *_when rate of self-fertilisation is set to 0.9. Click here for file

Additional file 7 **SNP ascertainment bias**. A PDF file with supplementary figure for SNP ascertainment bias in the four different geographic regions and the Nordborg dataset. Click here for file
