# Correction: Influence of mutation rate on estimators of genetic differentiation - lessons from *Arabidopsis thaliana*

**DOI:** 10.1186/1471-2156-11-88

**Published:** 2010-10-07

**Authors:** Ilkka Kronholm, Olivier Loudet, Juliette de Meaux

**Affiliations:** 1 Department of Plant Breeding and Genetics, Max-Planck Institute for Plant Breeding Research, Carl-von-Linné-Weg 10, 50829 Cologne, Germany; 2 Institut Jean-Pierre Bourgin, UMR1318 INRA-AgroParisTech, F-78000 Versailles, France

## Abstract

It has been brought to our attention that our paper (Kronholm et al. BMC Genetics 2010, 11:33) may have caused some confusion for readers interested in the correct quantification of population differentiation. We feel that this issue is of some importance and wish to clarify any confusion that might have resulted.

## Correction

In the introduction of Kronholm et al. [1], we discuss what properties a differentiation measure, like *F_ST_*, should or was assumed to have. Recent developments [2-5] have shown that *F_ST _*in fact does not have these properties. Our intention was to take a chronological approach, referring to *F_ST _*as it has traditionally been referred to and subsequently emphasizing some of the problems this measure has.

Genetic differentiation among populations, that is differences in allele frequencies, is caused by multiple factors, demographic factors (genetic drift, migration etc.) and mutations.

Our goal was to identify which measure should be used when only the demographic parameters are of interest. This is the case when genetic divergence is examined in order to detect local adaptation and assess the ecological relevance of natural variation. In this case **Φ**_*ST *_can be useful, if different markers need to be compared to each other. Given this context, this is why we state that the measure should be independent of mutation rate.

On the other hand, if one is interested in the question: is there genetic differentiation *per se *among the studied populations? Then, a measure like *D *should be used because it measures the absolute genetic differentiation present. We do not wish to advocate in general that a measure of genetic differentiation should be independent of mutation rate.

In some places of our paper this message does not come through clearly, because our phrasing may be less than ideal. Also, sometimes the word "estimator" was used when "measure" would have been a better choice. We do not wish to say for example that *D *is a "statistical estimator" of *F_ST _*but that these are different measures of genetic divergence.

Furthermore, we wish to address table 1, where the expected value of *F_ST_*, and values for *D *and *F'_ST _*are given in the same table. Here, it was not stated clearly enough that values of *D *and *F'_ST _*cannot be directly compared to the expected *F_ST _*value. The idea here was to show how the different measures depart from the expected *F_ST _*which is sensitive only to demographic parameters. It is not meant to imply that *D *or *F'_ST _*perform "poorly".

We offer our sincere apologies to the community for any confusion we may have caused.
