# Supplementary material for: Influence of mutation rate on estimators of genetic differentiation - lessons from Arabidopsis thaliana
Source: BMC Genet. 2010 May 1;11:33. doi: 10.1186/1471-2156-11-33 (PMC2888750; doi:10.1186/1471-2156-11-33)
Supplement: Additional file 5 — Computer simulations with selfing. A PDF file with a supplementary figure for results of computer simulations when the rate of self-fertilisation is set to 0.9. [file 1471-2156-11-33-S5.PDF]

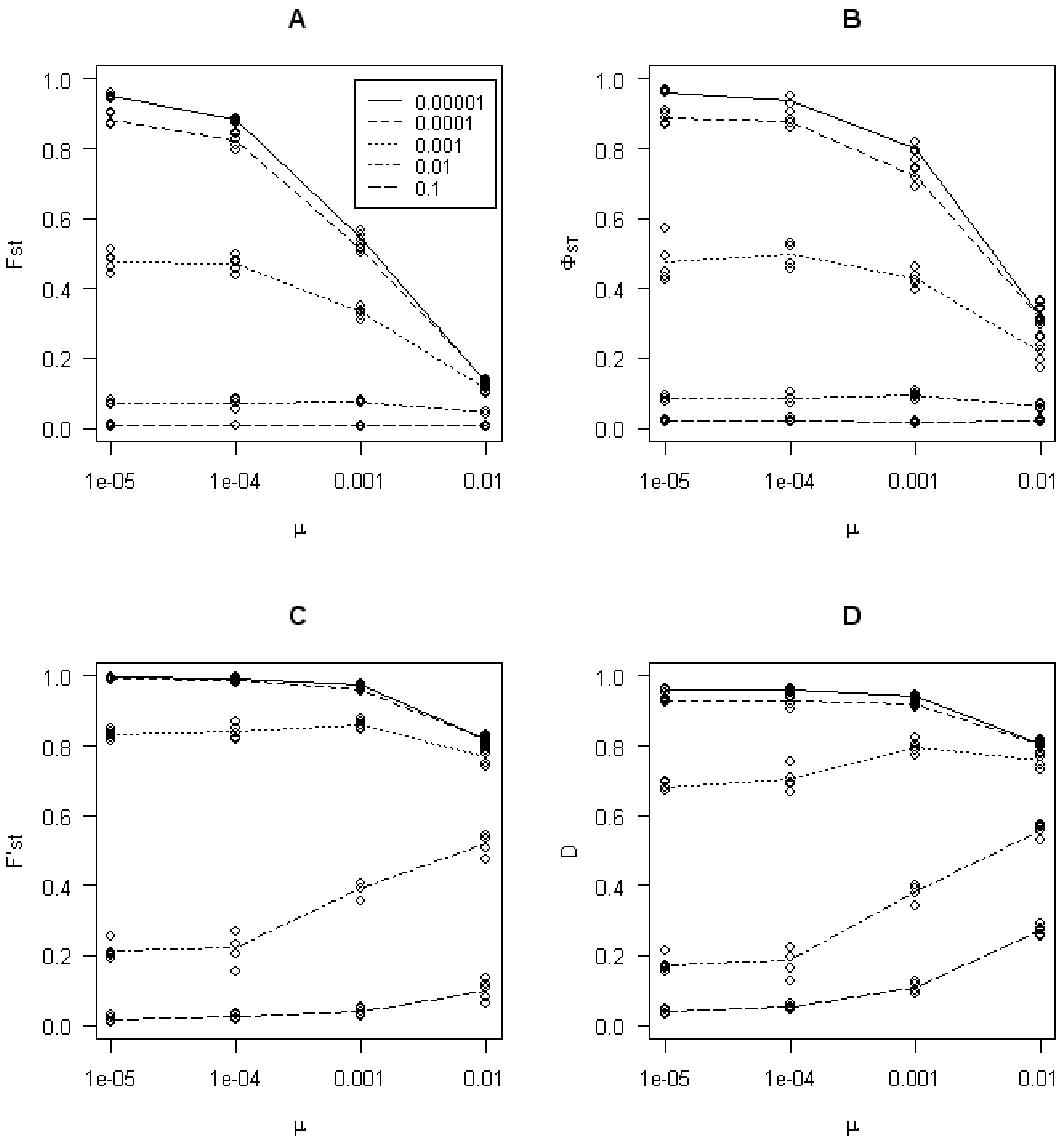

Figure S1. Results of forward simulations for self-fertilisation rate 0f 0.9. The mixed mutation model was used. Shown are the relationships with different estimators of genetic differentiation to different migration and mutation rates. X-axis is the mutation rate; different types of lines correspond to different migration rates as in the legend in panel A. Different estimators are F_{ST}, Φ_{ST}, F'_{ST} and D in panels A, B, C and D respectively.
