# Supplementary material for: Influence of mutation rate on estimators of genetic differentiation - lessons from Arabidopsis thaliana
Source: BMC Genet. 2010 May 1;11:33. doi: 10.1186/1471-2156-11-33 (PMC2888750; doi:10.1186/1471-2156-11-33)
Supplement: Additional file 6 — Computer simulations for QST with selfing. A PDF file with a supplementary figure for results of computer simulations for QST when rate of self-fertilisation is set to 0.9. [file 1471-2156-11-33-S6.PDF]

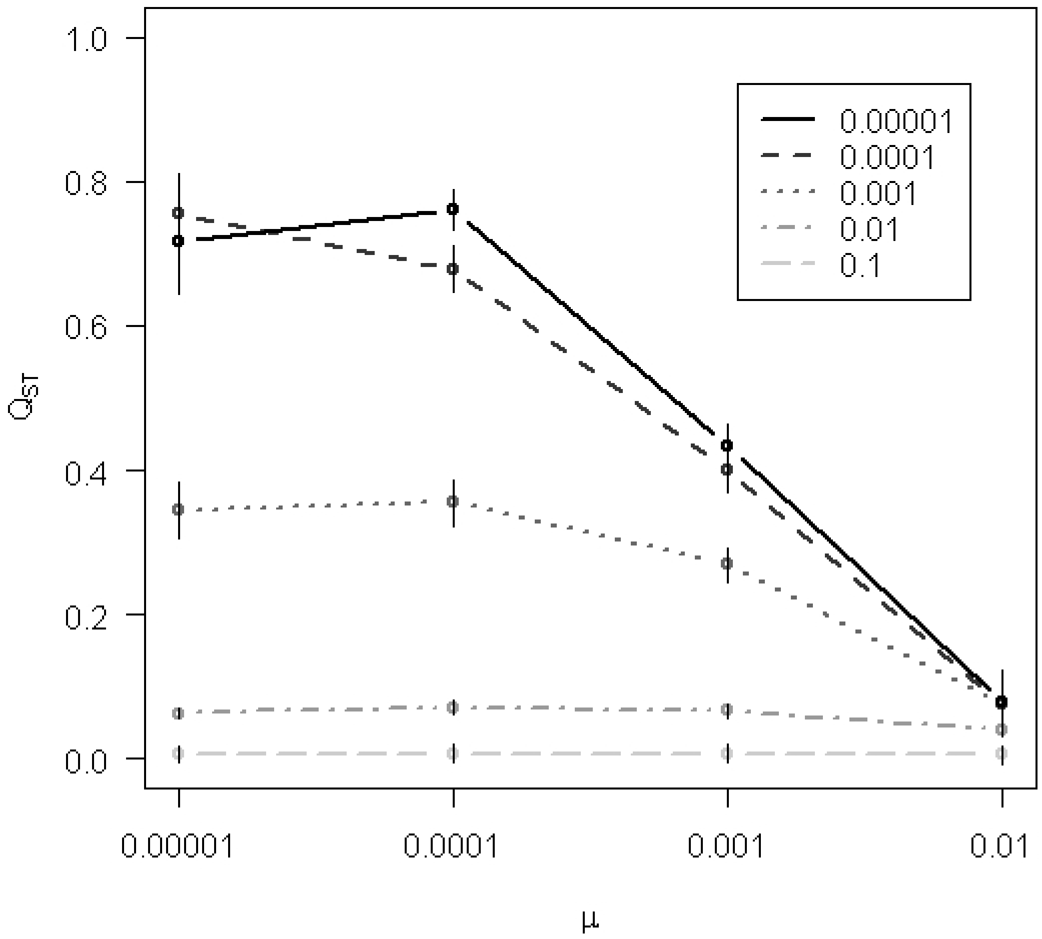

Figure S2. Results of computer simulations for Q_{ST} with rate of self-fertilisation set to 0.9. Migration rates 0.1, 0.01, 0.001, 0.0001 and 0.00001 correspond to different lines as indicated by the legend. Points are means of 50 replicate simulations and vertical lines correspond to 95 % confidence intervals.
