# Supplementary material for: Influence of mutation rate on estimators of genetic differentiation - lessons from Arabidopsis thaliana
Source: BMC Genet. 2010 May 1;11:33. doi: 10.1186/1471-2156-11-33 (PMC2888750; doi:10.1186/1471-2156-11-33)
Supplement: Additional file 7 — SNP ascertainment bias. A PDF file with supplementary figure for SNP ascertainment bias in the four different geographic regions and the Nordborg dataset. [file 1471-2156-11-33-S7.PDF]

A

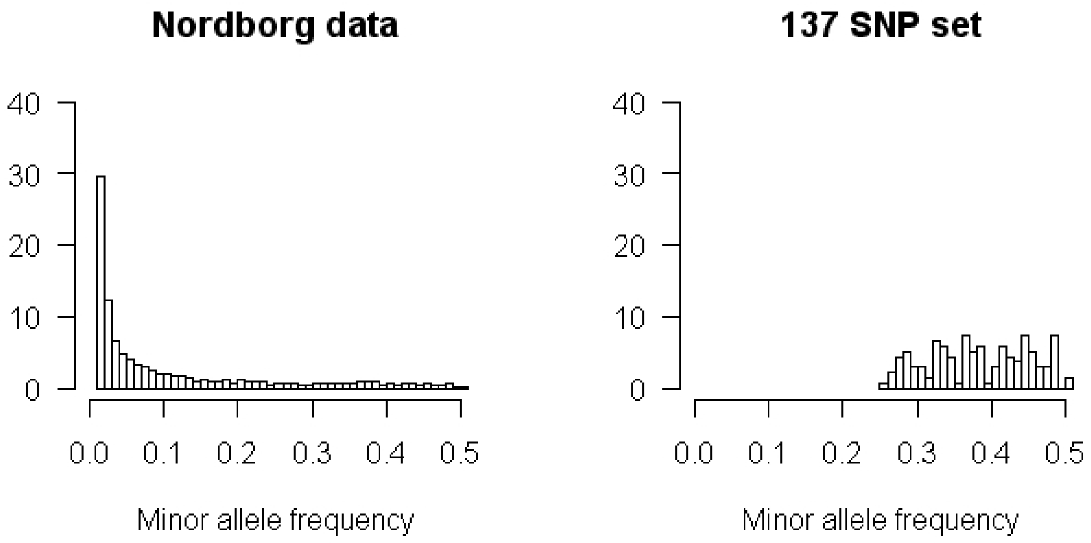

B

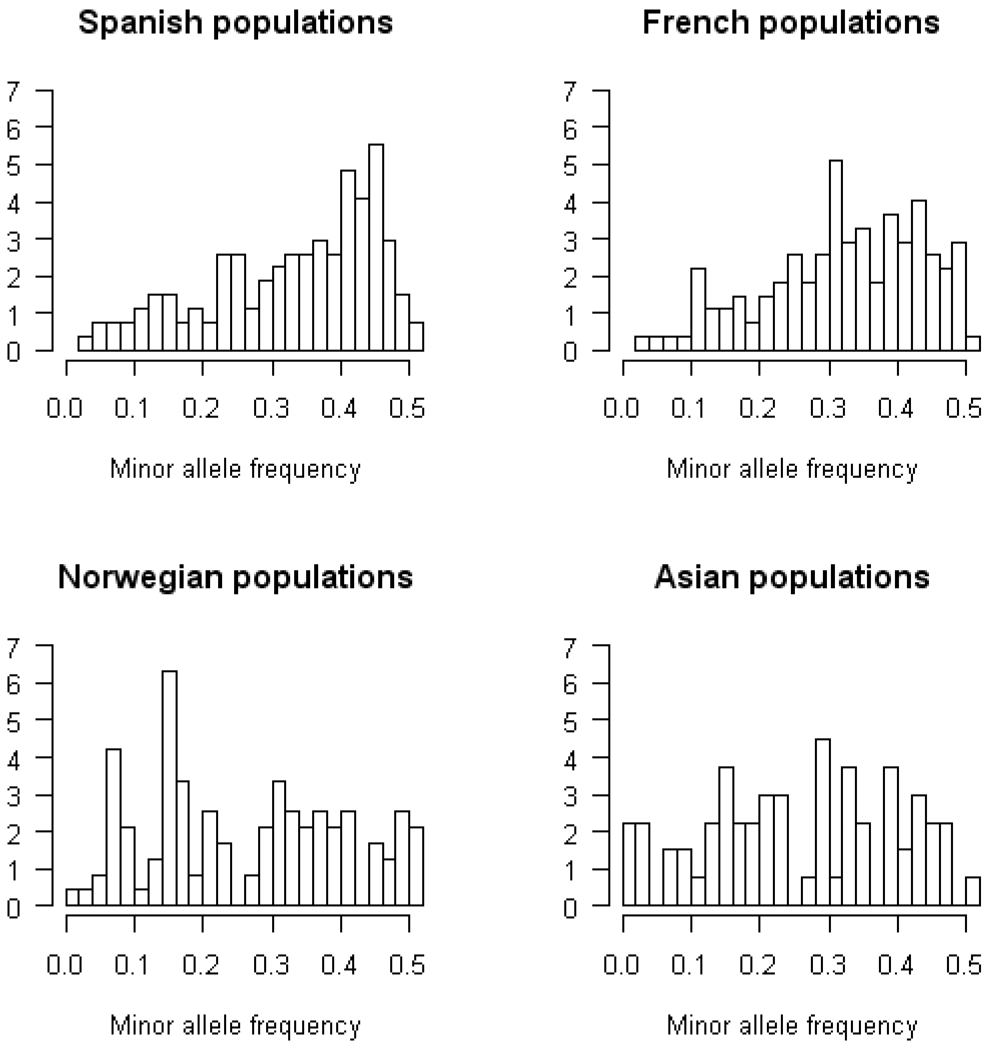

Figure S3. A) Minor allele frequency plots for the whole Nordborg data, and using only the 137 SNP markers used in our study. As it can be seen 137 SNP set excludes all rare SNPs and

has an excess of common SNPs. B) SNP minor alleles frequency distributions for each region. Note that in both panels y-axis has relative frequency density scale, so that the area under the histogram equals one.
